# Evaluating the Effects of Non-Neutral Molecular Markers on Phylogeny Inference

**DOI:** 10.1371/journal.pone.0087428

**Published:** 2014-02-18

**Authors:** Dawn M. Roje

**Affiliations:** Department of Ichthyology, American Museum of Natural History, New York, New York, United States of America; CNR, Italy

## Abstract

Nucleotide substitution models used in molecular phylogenetics do not account for nucleotide sequences evolving under selection, yet selection is rarely tested for. If non-neutral markers violate these models (i.e. non-independence of sites), it is expected that their reconstructed topologies be incongruent with those inferred from neutral ones and conclusions made from those phylogenies should be reexamined. Using rhodopsin as a phylogenetic marker has recently been called into question for exactly this reason. Rhodopsin is assumed to have evolved under strong positive selection for organisms that inhabit similar aquatic environments, making it unsuitable for the phylogenetics of aquatic organisms, but it is unclear what the effects of non-neutrality on phylogeny estimation are. To evaluate potential incongruence of neutral versus non-neutral markers, and the notion that rhodopsin should not be used in the molecular phylogenetics of fishes, a molecular dataset of 78 acanthomorph taxa and sequences from four nuclear, protein coding loci (including rhodopsin), were examined. Only one marker was found to be neutral while the remaining tests, for all other loci, rejected the null hypothesis of neutrality. To evaluate the possible effect(s) of positively versus negatively selected sites, the three non-neutral markers were analyzed to determine the presence of positively and negatively selected codons. To determine congruence in topology among ML trees inferred by individual neutral and non-neutral markers, as well as the combined (concatenated) dataset, tree, comparisons of distances among trees and hypothesis (topology) testing were carried out. Results of the tree distance metrics and topology testing support the notion that neutrality alone does not determine congruence in topology, and those data that are inferred to have evolved under selection should not necessarily be excluded. In addition, the number of sites inferred to have evolved under positive selection does not predict congruence with other markers or the topology inferred with the concatenated dataset.

## Introduction

There exist a large number of genes used to infer phylogeny of which most substitution models describing their evolution are independent, finite sites models implicitly assuming neutrality. Although violations of these assumptions should lead to unsound phylogeny inference, this has not been tested on a real dataset. If the evolution of sites is correlated, as they would be for markers that have evolved under strong pressures of natural selection, convergent molecular evolution at those sites can lead to erroneous inference of phylogeny and should be inconsistent with the phylogenetic signal inferred from neutral ones. In fact, non-neutral convergent molecular evolution has been detected in mitochondrial genes and has been shown to cause problems in phylogenetic inference [1], namely that the mtDNA phylogeny is incongruent with the more robust phylogeny inferred from nuclear DNA. The practice of testing for selection regardless of species gene tree incongruence, however, is almost nonexistent. Is it highly unlikely that all of the numerous molecular markers sequenced for various taxonomic groups are all neutral. If they are not, and selection does negatively affect phylogenetic inference (either by violating assumptions of the models or representing convergent evolution) then all of the conclusions made from those studies would be untrustworthy. Many would argue that those studies should be trusted because sequences from multiple genes were (usually) analyzed, and that when multiple independent lines of evidence are analyzed they are anticipated to converge on one hypothesis.

One way of testing this notion is to compare inferred phylogenies with the correct, true tree. The only way a topology can be considered correct is if it is derived through simulation. Hagstrom et al. [2] and Hang et al. [3] carried out such simulation studies and determined that if selection occurred between nodes, parsimony and neighbor-joining methods were not only able to recover the true tree but their performance was improved. These studies, however, only examined the reconstruction of four taxon statements—a trivial number of taxa when compared to those used in most phylogenetic studies.

Recently, Larmuseau et al. [4] investigated the effects of a marker, rhodopsin1 (referred to in their study as *rh1* and in others as well as this study as *rho*), under selection on the phylogeny estimation of a real group of organisms: the sand gobies of the teleost fish family, Gobiidae. They determined that similarities in *rho* sequences were due to convergence of species inhabiting similar light environments by linking functional regions of the protein to particular photic habitat and by constructing and comparing phylogenies based on *rho* and the ‘neutral’ genes, 12S and 16S. They determined that “The discordant patterns between the ‘neutral’ and the RH1 phylogeny indicate that rhodopsin is not an optimal marker for phylogeny reconstruction of aquatic taxa. Therefore, it is recommended to avoid the use of rhodopsin as a ‘neutral’ marker in phylogenetic studies,” (p. 695). If the assertions of Larmuseau et al. [4] are correct, conclusions made from the many fish phylogenies in which this marker has been used as part of a “total-evidence” (concatenated dataset) approach [5]–[10] should be considered suspect. However, tests for neutrality were only carried out on the *rho* sequences, ignoring the possibility that selection could also be detected for the supposed neutral *12s* and *16s* sequences. Additionally, although the ‘neutral’ and *rho* phylogenies appeared to be incongruent, no tree distance metrics were generated and no hypothesis testing was carried out.

Larmuseau et al.'s [4] claim that phylogenetic signal provided by rhosopsin is incongruent with different markers has been corroborated by others working on goby phylogeny [5], [6]. Tornabene et al. [6] noted that the tree inferred from *rho* data was different from the others, but did not test any of the markers for selection, nor did they test whether the difference in topologies were statistically significant. Because of this they could not prove or disprove Larmuseau's et al.'s [4] assertion that unrelated goby species inhabiting similar photic enviormnments will converge on similar *rho* sequences. Niemiller et al. [5] did test for selection in *rho* and rejected the null hypothesis of neutral evolution in certain goby lineages. These tests, however, were only carried out for *rho* and they did not evaluate any of the other individual markers for topology congruence. It is the goal of this study to test for topological incongruence between neutral and non-neutral nuclear markers, as well as to test the specific notion that *rho* is an inappropriate marker for the phylogeny reconstruction in fishes.

## Materials and Methods

Tests of selection are based on assessing the ratio of synonymous to non-synonymous nucleotide substitutions, therefore, only protein coding loci were chosen for this study. To test the notion that rhodopsin may not be an appropriate marker for phylogenetic analysis of aquatic organisms [4], the acanthomorph dataset of Li et al. [11] was selected. Those data include ring finger protein 213 (*rnf213*), mixed-lineage leukemia (*mll*), inverted repeat binding protein (*irbp*) and rhodopsin (*rho*) sequences; the former was sequenced for Li et al.'s (2009) study, the *rho* sequences were generated by Chen et al. [7], and the *mll* and *irbp* sequences (with the exception of a few sequenced by Li et al. [11]) were generated by Dettai and Lecointre [8]. Only taxa with accessioned sequence data for all four markers were included. Of those, *Epinephalus aeneus* was excluded because the published *irbp* sequence (AY362227) BLASTed [12] against the NCBI nucleotide database (with default parameters) as *mll*. All sequences used in this study were retrieved from GenBank.

All four markers were aligned using MUSCLE [13] implemented in Geneious (Biomatters Ltd., Auckland, New Zealand) with full penalty for terminal gaps, a gap open score of −1, and a maximum of eight refinement iterations. Model selection, utilizing the AICc (corrected Akaike Information Criterion), and phylogeny inference, using the ML criterion, were carried out in Treefinder [14]. Five alignments were analyzed, comprising each of the four individual markers and a concatenated dataset.

To test for selection, the Nei-Gojobori method (Proportion), implemented in MEGA 4.0 [15] was used to estimate the number of non-synonymous substitutions per non-synonymous sites (dN) and the number of synonymous substitutions per synonymous site (dS) for each of the four markers. Variances were generated by bootstrapping (5000 replicates) the data and then the null hypothesis of neutral evolution (H_0_: dN = dS) was tested using a Z-test covering the overall average (per marker) of dN and dS.

To detect the type of selection (positive or negative) per site the fixed effects likelihood (FEL) and the random effects likelihood (REL) methods were implemented in HyPhy [16], [17]. Both methods are based on ML estimates for the parameters of a nucleotide substitution and codon model, testing whether dN/dS >1 per site. Unlike REL, FEL estimates are conditional on a specific phylogeny; for these FEL analyses the TE phylogeny was used. The REL method assumes distributions for the synonymous and nonsynonymous rates and identifies positively selected sites using empirical Bayes factors. To determine significance, a cutoff p-value of 0.05 was used for FEL and an acceptance ratio of 0.05 for REL.

Tree distance metrics were generated and statistical tests were carried out on all five (four individual markers plus the concatenated dataset) topologies to assess their pairwise similarity or difference. Two tree distance metrics were used to evaluate topology congruence: the symmetric distance of Robinsons and Foulds [18], carried out in Phylip [19], and the SPR (subtree pruning and regrafting) heuristic distance [20], implemented in TNT [21]. The Symmetric Difference measures how many partitions are on one tree and not the other, whereas SPR distance measures the minimum number of SPR moves required to transform one tree into another. For both tree metrics, trees were treated as unrooted and for SPR distances, 2000 replications per comparison were carried out.

Because a tree metric is not a statistical hypothesis test, three paired sites tests, the KH [22], AU [23] and SH [24] tests were carried out. All tests compared likelihood differences among tree topologies (the five generated for this study) to the empirical variation in log likelihoods for a given dataset, with the AU and SH tests approximately correcting for multiple trees. The null hypothesis (H_0_: all topologies for comparison are equally good explanations of the data) was rejected when P<0.05. The hypothesis tests were implemented in Treefinder [14] using the RELL (resample estimated log-Likelihood) nonparametric bootstrap method for the KH and SH tests with 50,000 replicates to generate the null distributions. A multiscale bootstrap technique was used for the AU test, which is considered to exhibit the least amount of bias and is less conservative than the SH test [14]. The models used to calculate the likelihoods for each topology were the same as those chosen using the AICc in Treefinder [16].

To determine if incongruence was caused only by the presence of positively selected codons, any marker that was found to be incongruent had its positively selected sites (all those sites detected using FEL and REL) removed and another round of KH, AU and SH tests was carried out in Treefinder [14]. If sites under positive selection are the only cause of incongruence, their removal should result in failure to reject the null hypothesis of the paired sites tests.

## Results

The aligned matrices consisted of 78 OTUs with the following lengths per marker: 991 bp (603 informative sites) of *rnf213*, 542 bp (364 informative sites) of *mll*, 716 bp (488 informative sites) of *irbp*, and 759 bp (404 informative sites) of *rho*. The final concatenated, TE, dataset consisted of 3008 bp (1859 informative sites). All four alignments were straightforward in that no internal stop codons were detected and all indels corresponded to one or more codons.

Using the AICc, Treefinder [14] indicated that the best-fit nucleotide substitution model (given a parameter penalty and correction for sample size) for the combined dataset (all four genes) is GTR+G. Individually, using the same approach, the following models were chosen: HKY+G for *rho*, J2+G for *irbp* and GTR+G for *mll*; and *rnf213*; those models were used to generate the ML trees. The maximum (negative) log-likelihoods, determined by Treefinder [12], for each of the five trees were as follows: −18045.83 for *rnf213*, −11770.489 for *mll*, −17056.246 for *irbp*, −14702.74 for *rho* and −62719.57 for the tree inferred from the concatenated dataset (from now on referred to as the “Con” tree or topology). The ML trees inferred from the concatenated dataset, as well as each individual marker, with 1000 bootstrap replicates is available in File S1.

The overall test of selection resulted in the following p-values: 0.639 for *rnf213* and 0.000 for the remaining three markers. *Rnf213* was the only marker that failed to reject (P<0.05) the null hypothesis (H_0_: dN = dS) of neutral evolution; all others can be treated as having evolved under some pressure(s) of selection.

The results of the tree distance comparisons are summarized in Table 1 (symmetric differences) and Table 2 (SPR distances). The shortest distances of all pairwise comparisons were that of *rnf213* (the neutral marker) to the Con tree (symmetric distance of 70 and SPR distance of 19). The *irbp* tree was furthest from the Con tree with a symmetric distance of 110 and an SPR distance of 46 or 47. Comparisons among the individual (inferred from one marker) topologies resulted in symmetric distances ranging from 98 (*rnf213*/*mll*) to 122 (*irbp*/*rho*) and SPR distances ranging from 36, 37 (*rho*/*mll*) to 61, 63 (*irbp*/*rho*). The was no clear pattern indicating that the non-neutral markers (*mll*, *irbp* and *rho*) were further from the neutral one (*rnf213*) than either was to each other. For example, the symmetric distance from *rnf213* to *rho* was 112, but the distance from *mll* to *irbp* was the same. *Rho* was the furthest from the neutral marker, but those distances (symmetric and SPR distances) were not greater than the distances from *rho* to *irbp*.

**Table 1:** Pairwise Symmetric Distances between topologies; non-neutral markers are in boldface.

	*rnf213*	*mll*	*irbp*	*rho*	Concatenated
*rnf213*	—	98	110	112	70
***mll***	98	—	112	120	88
***irbp***	110	112	—	122	110
***rho***	112	120	122	—	102
Concatenated	70	88	110	102	—

**Table 2:** Pairwise SPR distances.

	*rnf213*	*mll*	*irbp*	*rho*	Concatenated
*rnf213*	—	41	48	48	19
***mll***	40	—	49	36	41
***irbp***	50	43	—	63	47
***rho***	51	37	61	—	42
Concatenated	19	41	46	45	—

First number in cell represents the distance from the tree in the left column to the tree in the top row; the second number is the distance computed from the reverse direction; non-neutral markers are in boldface.

The symmetric and SPR distances were not entirely consistent in rank; they differed in the tree distances from *mll* to *rho* and vice versa. The symmetric distance between *mll* and *rho* was 120, the second largest distance among all pairwise symmetric distances comparisons. The SPR distances, however, had *mll* and *rho* 36 or 37 steps away from each other, the second smallest SPR distance among those comparisons.

Tree distance metrics cannot be used to reject the hypothesis that two trees are not significantly different. Because of this, KH, AU and SH tests were conducted on differences in log-likelihoods of the five topologies and datasets; the results are summarized in Table 3.

**Table 3:** Results of the KH, AU and SH tests.

Data	Tree	KH Test (P-value)	AU Test (P-value)	SH Test (P-value)
*rnf213*	*rnf213*	—	—	—
	*mll*	0.000	0.000	0.000
	*irbp*	0.000	0.000	0.000
	*rho*	0.000	0.000	0.000
	**TE**	**0.220**	**0.222**	**0.661**
*mll*	*rnf213*	0.000	0.000	0.003
	*mll*	—	—	—
	*irbp*	0.000	0.000	0.000
	*rho*	0.000	0.000	0.000
	**TE**	**0.058**	**0.057**	**0.198**
*irbp*	*rnf213*	0.001	0.000	0.003
	*mll*	0.000	0.000	0.000
	*irbp*	—	—	—
	*rho*	0.000	0.000	0.000
	**TE**	**0.111**	**0.098**	**0.345**
*rho*	*rnf213*	0.000	0.000	0.000
	*mll*	0.000	0.000	0.000
	*irbp*	0.000	0.000	0.000
	*rho*	—	—	—
	TE	0.000	0.000	0.000
Concatenated	*rnf213*	0.000	0.000	0.000
	*mll*	0.000	0.000	0.000
	*irbp*	0.000	0.000	0.000
	*rho*	0.000	0.000	0.000
	TE	—	—	—

Boldface rows represent those tree topologies that are not significantly different from the phylogeny with the best likelihood score.

All three paired sites tests were consistent in their rejection (P<0.05) or acceptance (P>0.05) of the null hypothesis, with the SH test being the most conservative. In all pairwise comparisons, except for the *rnf213*/Con, *mll*/Con and *irbp*/Con comparisons, the hypothesis that the topologies are explained equally well by the data was rejected. Of the three that failed to reject, the largest p-values were associated with comparison of *rnf213* to the Con tree. All tests indicate that the *rho* tree is significantly different from not only all other individual marker trees, but also the Con tree.

Of the three non-neutral markers, *rho* had the most sites where positive selection was detected (see Table 4). Two sites (codon positions 121 and 165) were detected by FEL and four sites (codon positions 118, 121, 165 and 169) were detected by REL. Because the two sets of sites overlap, all four codons detected by REL were removed from the Rho alignment that was then used in three paired sites test to determine congruence with the *rnf213*, *irbp*, *mll* and Con topologies. All four tests resulted in p-values of 0.000, rejecting (P<0.50) the null hypothesis that the topologies for comparison are equally good explanations of the modified *rho* data.

**Table 4:** Summary of results from the FEL and REL analyses of the three non-neutral markers.

	FEL		REL	
	Positive	Negative	Positive	Negative
*irbp*	1	179	0	185
*mll*	1	150	1	158
*rho*	2	168	4	193

## Discussion

It is important to remember that the Con topology is not (necessarily) the “true tree”, but the sum of the contributions of the four individual datasets. For this reason, the closeness in tree distance of *rnf213*, the neutral marker, to the TE phylogeny can be attributed to the proportion of data it contributed to the concatenated dataset (603 of 1859 informative sites); that amount being the greatest contribution of all four markers. Therefore, comparisons of topology congruence of the trees inferred by the individual markers will be the focus of this discussion, in that they are better indications of the behavior of non-neutral versus neutral markers.

Both tree distance metrics show that the amount of shared partitions and number of SPR moves to edit one topology into the other is no shorter or longer among the non-neutral markers than they are to *rnf213*. This is easily seen upon examination of the distances of the *irbp* topologies to all others. Both metrics have the *irbp* topology farthest from the *rho* tree and both markers failed the Z-test of selection (H_0_: dN = dS). Also, the closest distance between individual marker topologies was that between *rnf213* (neutral) and *mll* (non-neutral), with a symmetric difference of 88. *Rho* and *irbp* are closer to *rnf213* than they are to each other. This lack of clustering of topologies inferred from non-neutral markers supports the notion that non-neutrality alone does not warrant the omission of these data from phylogenetic analyses.

The hypothesis tests described above provide additional support for the inclusion of non-neutral molecular data in phylogeny inference. The p-values for the *rnf213*, *mll* and *irbp* data given the Con topology were all above 0.05, indicating that the Con topology is an equally good explanation of neutral as well as non-neutral data. It is worth noting, however, that *rnf213* had the greatest p-values, but, just as with the tree distance metrics, this can be attributed to its large contribution to the concatenated dataset.

The *rho* data had the third largest contribution of informative sites to the combined concatenated dataset but was significantly different from all other topologies. This, combined with the incongruent behavior of the *rho* topology in the tree distance comparisons, supports the assertion of Larmuseau et al. [4] that phylogenetic signal provided by Rho is disparate from that of other markers. Although, it is important to remember that this is only in one case. To make these conclusions more generalizable it would be necessary investigate the effects of *rho* in many different groups of aquatic organisms and compare those results with that of terrestrial taxa. The fact that the Rho data are incongruent with both neutral *and* non-neutral topologies, however, allows for the rejection of Larmuseau et al.'s [4] claim that this difference is due to natural selection since selection alone was not able to predict congruence. In addition, the presence of positively selected sites in the Rho alignment does not predict phylogenetic incongruence. If transformations at those sites are homoplasies then the signal they provide is misleading, but their removal does not make Rho congruent with the other makers or the Con topology—something else has contributed to the unique evolutionary history of this marker. Of the four markers evaluated here, *rho* is the only G-protein coupled receptor (GPCR); it would be valuable to compare the signal provided by GPCR markers to those from other protein families.

Incongruent signal provided by different loci is precisely why the practice of molecular phylogenetics requires the use of multiple unlinked markers. It is expected that that any misleading information, such as convergence, will be ameliorated by the remaining data, which is exactly what occurred here. Although the phylogenetic signal of Rho was inconsistent with the other markers, it was also inconsistent for the Con topology, which is usually regarded as the most favorable phylogeny. This shows that the mere state of being neutral or non-neutral does not warrant exclusion of data. This case study, while highlighting the potential problems with the use of a particular marker on phylogeny estimation also shows that the common practice of using multiple markers can mitigate its potentially deleterious effects.

## Supporting Information

File S1 **Nexus tree files with bootstrap support for all five trees presented; Con,** ***rnf213*** **,** ***mll*** **,** ***irbp*** **and** ***rho*** **.**
(ZIP)
